# Zombies without puppeteers: Morgan’s Canon and the passive manipulation hypothesis in *Xenos vesparum*/*Polistes dominula*

**DOI:** 10.3389/fpara.2026.1907843

**Published:** 2026-09-03

**Authors:** Philip T. Starks

**Affiliations:** Department of Biology, Tufts University, Medford, MA, United States

**Keywords:** active manipulation, behavioral parasitology, extended phenotype, host behavior, passive manipulation, *Polistes dominula*, zombie parasites, Xenos vesparum

## Abstract

Parasite-associated changes in host behavior are often framed as manipulation, especially when altered behavior benefits parasite transmission. In the *Xenos vesparum*/*Polistes dominula* system, infected wasps abandon nests, aggregate before hibernation, overwinter, and later join established nests; a sequence often described through zombie or puppet-master metaphors. Recent studies have tied this phenotype to caste-related plasticity, gene expression, neuroanatomy, and ecological context, but the causal distinction between active parasite control and passive activation of host responses remains unresolved. Here I argue that passive manipulation should be treated as a useful null model and possible evolutionary starting point: infection may trigger pre-existing host responses that the parasite subsequently exploits. Archived pseudo-parasite observations are presented as an illustration: a sterile nylon implant, with no capacity for signaling, was associated with reduced energy use and nest desertion, whereas encapsulation intensity did not predict metabolic change. These observations do not prove passive manipulation by *X. vesparum*, but they show that early components of the infected phenotype can be induced without a living parasite. More broadly, parasite-benefiting behavior should not be treated as sufficient evidence for parasite-designed control. Distinguishing active from passive manipulation requires experiments that compare true infection, mechanical insult, parasite secretions, and naturally occurring host alternative phenotypes.

## Introduction

The experiment began with a simple question: what does it cost a wasp to fight off a parasite? Two students - one working on insect energetics, one on immunology - joined forces in my lab to find out. We implanted sterile nylon monofilaments into the abdomens of *Polistes dominula* workers, approximating the physical presence of the strepsipteran *Xenos vesparum*, and measured the metabolic consequences of mounting an encapsulation response. We expected elevated CO2 production after implantation. The wasps did something else. Implanted individuals tended to down-regulate energy use, and in a field enclosure, they were also more likely to leave their nests. We had not set out to study parasitic manipulation, but we had stumbled onto the edge of it.

That question sits on one of the more theatrically compelling systems in behavioral parasitology: the *X. vesparum/P. dominula* relationship (Moore, 2002). Parasitized workers become sluggish, abandon their nests, aggregate at pre-hibernation sites, overwinter, and emerge the following spring to join established colonies, where the parasite can infect developing brood (Hughes et al., 2004; Beani, 2006; Beani et al., 2011). The parasite reproduces sexually in these aggregations (Hughes et al., 2004; Beani et al., 2005).

The narrative writes itself: a parasite turns a social wasp into a vehicle for its own transmission. The word “zombie” is almost irresistible, and popular coverage has frequently framed these parasites as “outsider zombie queens” (Keim, 2011). However, Doherty (2020) has cautioned against the risks of this narrative, noting that such anthropomorphic language can obscure the underlying biological complexity of host-parasite interactions.

This is a critical concern, as distinguishing between the by-products of infection and active parasitic manipulation is a long-standing challenge in the field. For three decades, researchers have grappled with this problem, recognizing that altered host behavior can often be misinterpreted as adaptive manipulation when it is actually a pathological by-product or a host-mediated response to infection (Poulin, 1995; Cézilly and Perrot-Minnot, 2005; Thomas et al., 2005; Liu et al., 2020). Furthermore, it has been established that parasitic manipulators may simply exploit existing host circuits rather than inventing new behavioral programs (Adamo, 2013).

Here, I argue that the zombie narrative risks outrunning the evidence for its central mechanistic implication. I propose that passive manipulation, a host response triggered by infection that coincidentally benefits the parasite, should be treated as a useful null model and a possible evolutionary starting point for this system. If the host already has the behavioral architecture, the parasite may not need to build it. It may only need to activate it.

## From zombie queens to caste plasticity

The framing of this system has not stood still. Early accounts emphasized the striking fit between the parasite’s life cycle and the altered behavior of infected hosts: parasitized wasps desert colonies, aggregate, overwinter, and later visit nests in ways that facilitate parasite mating and transmission (Hughes et al., 2004; Beani et al., 2011). That framing made sense, but it also encouraged a shorthand in which parasite-benefiting behavior became manipulation by default.

More recent work has moved the field toward a subtler picture. Transcriptomic analysis showed that stylopized workers exhibit gyne-shifted brain expression patterns, suggesting that *X. vesparum* affects its host by exploiting phenotypic plasticity related to social caste (Geffre et al., 2017). Long-term observations on trumpet creepers placed the system in an ecological context in which altered spatial and feeding behavior can benefit both host survival and parasite transmission, leading the authors to emphasize co-evolved or shared phenotypes rather than simple takeover (Beani et al., 2018). Neuroanatomical work further showed that caste, sex, and parasitism interact in shaping brain-region allocation, again tying the parasite-associated phenotype to pre-existing axes of wasp plasticity (Gandia et al., 2022).

That movement is good for the present argument. The strongest version of the passive manipulation hypothesis is not a rejection of the manipulation literature. It is the next step in its refinement. If the parasite-associated phenotype is built from caste-related plasticity, we still need to know whether the parasite actively directs the shift or whether infection activates a host program that was already available. Put differently, the field has moved from mind control toward plasticity; the next question is whether the parasite controls that plasticity or benefits from releasing it.

## Morgan’s Canon as a mechanistic caution

C. Lloyd Morgan’s Canon is usually invoked in discussions of animal cognition: do not interpret an action as the product of a higher faculty when a lower one is sufficient (Morgan, 1894). The same caution can be applied to mechanism. Before attributing post-infection behavior to a specialized parasite signal that reprograms the host nervous system, we should ask whether a simpler host-response mechanism is sufficient.

This is not an argument against adaptation. It is an argument against skipping levels of explanation. In parasite-host systems, the outcome can be strongly adaptive for the parasite even when the proximate mechanism is generic. A wasp may desert a nest because it is injured, immunologically challenged, or physiologically compromised. If that response happens to move the host into a context that favors parasite mating or transmission, the parasite benefits. But benefit is not mechanism.

The broader manipulation field has increasingly recognized this problem. Reviews have emphasized that molecular, neurochemical, or physiological differences between infected and uninfected hosts are not automatically evidence of parasite-designed control, and that the mechanistic gap between altered host state and parasite fitness remains a central obstacle (Herbison, 2017; Hughes and Libersat, 2019; van Roosmalen and de Bekker, 2024). This problem is especially acute when altered host behavior resembles a known host response to stress, injury, or infection.

More formally, the distinction at issue is between active and passive manipulation (Poulin, 1994, Poulin, 2010). Active manipulation implies that the parasite produces a specific signal - neurochemical, endocrine, immune, genomic, or otherwise - that directs host behavior in ways that serve parasite transmission. Passive manipulation implies something less elaborate: the physical act of infection is sufficient to elicit host behaviors that, by circumstance, also benefit the parasite. The host is not being steered like a puppet; it is responding to being compromised in ways that the parasite can exploit.

Passive manipulation is therefore not a weak version of active manipulation. It is a different hypothesis, a plausible evolutionary starting point, and a useful null model. It requires no novel host-control machinery in the parasite. It requires only a host response that already exists, a parasite positioned to trigger it, and transmission benefits that follow from the host’s response.

## A host repertoire awaiting activation

The strongest reason to take the passive manipulation hypothesis seriously in this system is that the behavioral components of the *Xenos*-associated phenotype are not unique to parasitized wasps. Early-emerging females in social wasps can leave their natal nests, aggregate, overwinter, and reappear as potential reproductives the following spring (Reeve et al., 1998; Starks, 2001). In *P. dominula*, some spring females do not initiate nests; instead, they adopt or usurp nests initiated by others (Starks, 1998, Starks, 2001). These behaviors have been interpreted as alternative reproductive tactics, not as parasite-induced pathologies.

This matters because *X. vesparum* does not need to create an entirely novel behavioral sequence if the necessary components already exist in the host repertoire. The parasite could benefit from pushing a worker into a state that resembles an early disperser, a future foundress, or a ‘sit-and-wait’ female (sensu Starks, 1998, Starks, 2001). Under that interpretation, the wasp’s behavior is not alien. It is contextually misplaced.

A reasonable objection is that documenting components of the sequence in parasite-free populations is not equivalent to documenting the full *Xenos*-associated sequence in a single unparasitized individual. That distinction is important. But it does not rescue the stronger zombie claim. The presence of these components in the normal behavioral repertoire shifts the burden of proof. The question is no longer whether parasitized wasps behave in ways that benefit *Xenos*. They clearly do. The question is whether *Xenos* actively constructs those behaviors or triggers a host program already available to be expressed.

## An illustrative pilot observation

**Figure 1:**
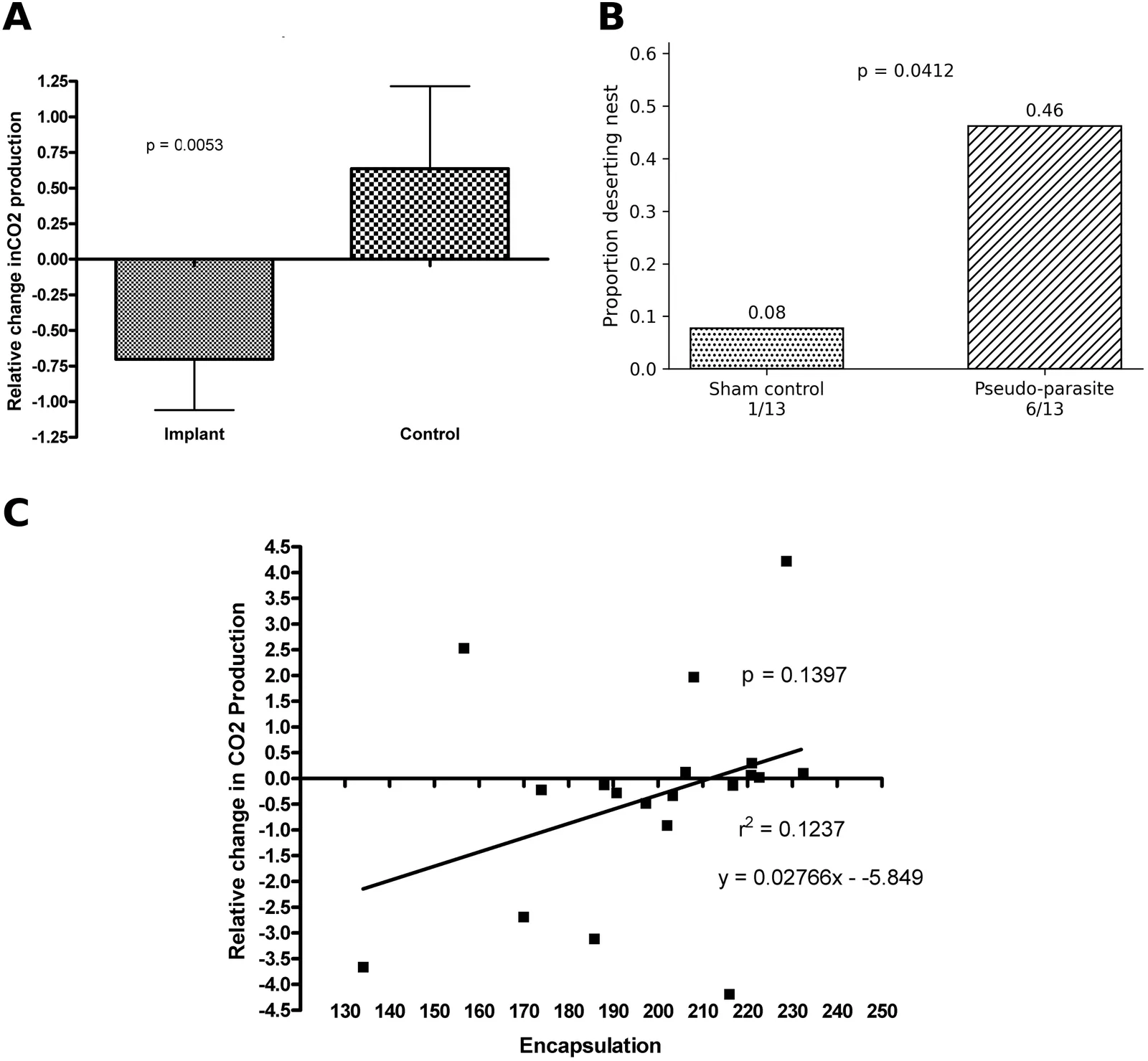
Archived and recovered pilot observations from pseudo-parasite implantation experiments. **(A)** Relative change in CO_2_ production after manipulation; negative values indicate lower post-manipulation CO_2_ production. The archived image reports p = 0.0053 for the contrast between implanted workers (n = 34) and sham-handled controls (n = 16). Separate archived analysis notes indicate that implanted workers showed a significant post-implantation decrease in absolute CO_2_ production (n = 34, t = 2.08, df = 33, p = 0.046) and a marginal decrease in relative CO_2_ production when considered alone (p = 0.059), whereas sham-handled controls showed no evidence of post-manipulation change (n = 16; archived p-values > 0.28). Because the underlying respirometry table is not presently available, this panel is presented as an archived illustrative pattern. **(B)** Nest desertion in the field enclosure. The surviving activity dataset confirms the plotted counts from 13 paired nests: pseudo-parasite, 6/13 workers deserted; sham control, 1/13 workers deserted. The archived presentation image reported p = 0.0412; the recovered activity table confirms the counts but not the original test file. **(C)** Encapsulation response and change in CO_2_ production. No relationship was detected between melanin deposition on retrieved monofilaments and relative change in CO_2_ production (n = 19; archived linear regression/correlation: p = 0.1397, r^2^ = 0.1237). The regression equation shown in the archived image is y = 0.02766x - 5.849. Because the underlying regression table is not presently available, this panel is presented as an archived pattern rather than a reanalysis.

The pseudo-parasite experiment mentioned earlier provides a useful illustration of what a passive trigger could look like. Workers were implanted with sterile nylon monofilaments 2 mm in length, inserted between the second and third sternites to approximate the physical presence of a protruding female strepsipteran. Sham-handled controls were anesthetized, weighed, prodded, and returned to their nests without implantation. Resting metabolic rate was measured before and after manipulation using differential open-flow respirometry (see Weiner et al., 2009; **Figure 1A**). Nest desertion was monitored in a field enclosure with 13 paired nests, each contributing one implanted and one sham-handled worker (**Figure 1B**). Encapsulation response was estimated from melanin deposition on retrieved monofilaments (see Wilson-Rich et al., 2014, Wilson-Rich et al., 2019; **Figure 1C**).

These observations should be read as hypothesis-generating rather than definitive. The archive is uneven: the surviving field-activity spreadsheet supports the nest-desertion counts, whereas the respirometry and encapsulation results are preserved as archived figures and analysis notes rather than full raw data tables. For that reason, the pilot observation should not be asked to carry the weight of a primary empirical paper. It is more useful as a proof of concept: an inert physical object, with no chemistry and no evolutionary history with the host, was sufficient to move wasps in the direction predicted by the passive manipulation hypothesis.

The pilot observations showed three patterns. First, implanted wasps showed lower relative CO2 production after manipulation than sham-handled controls in the archived figure (**Figure 1A**); separate analysis also indicates a post-implantation reduction in absolute CO2 production within implanted animals. Second, implanted wasps were more likely than controls to desert their nests in the enclosure, and this count is supported by the surviving activity dataset (**Figure 1B**). Third, the magnitude of the encapsulation response did not predict the change in CO2 production, suggesting that the metabolic shift was not simply a graded energetic cost of melanizing the implant (**Figure 1C**). Together, these observations do not prove that *X. vesparum* manipulates passively. They show that the first steps of the zombie-like phenotype can be produced without a living parasite.

## What the pilot observation can and cannot show

The most important limitation is also the most useful conceptual point: a sterile implant is not *X. vesparum*. It does not reproduce, feed, secrete parasite-specific compounds, or persist through the host’s seasonal transition in the way a true strepsipteran does. The pilot observation therefore cannot show that true infection lacks active components. It can only show that active parasite signaling is not necessary to produce two early features of the infected phenotype: reduced energy use and nest desertion.

A second complication is that the pseudo-parasite may have induced a generalized sickness response rather than a parasitism-specific syndrome. It is well-documented that injury, immune challenge, and infection across invertebrates can produce a suite of conserved sickness behaviors, including lethargy, anorexia, and social withdrawal (Sullivan et al., 2016). This broader context suggests that odd behavior in infected hosts is not automatically evidence of parasite-driven genetic control; rather, it may reflect the host’s physiological reaction to the presence of an intruder or the damage caused by it (Heil, 2016). That objection is fair, but it strengthens rather than weakens the passive manipulation hypothesis. If a broad, conserved response to physical insult is sufficient to produce behavior useful to the parasite, then the parasite may be exploiting a pre-existing host response rather than issuing specialized instructions.

A third complication is that passive and active manipulation are not mutually exclusive. A parasite could initially benefit from triggering a generic host response and later evolve mechanisms that amplify, prolong, or refine that response. Passive manipulation may be the ancestral condition from which active manipulation evolves. The point is not to replace one story with another. The point is to make the alternatives experimentally visible.

## A research agenda

Distinguishing passive from active manipulation requires experiments designed around mechanism, not merely around outcome. The most direct test would vary the site of implantation: if desertion and energy conservation depend on a specific anatomical interface - the location where a female *Xenos* actually protrudes between the tergites - then the response is at least consistent with a site-specific trigger. If the same behavioral changes follow from insult anywhere on the abdomen, passive triggering through a generalized injury response becomes the more parsimonious explanation. A complementary approach would separate wound response from the persistent presence of a foreign body, by comparing wasps given a puncture wound alone against those with a retained implant. If the implant’s continued presence is necessary to sustain behavioral change, something about chronic physical insult or chronic immune activation is doing the work. This would still be passive, but more specific.

The harder question is whether *Xenos* contributes anything chemical. Parasite extracts or candidate secretions applied without implantation would test whether a non-mechanical signal is sufficient, necessary, or irrelevant. That experiment is now more tractable than it once was: the transcriptomic work of Geffre et al. (2017) identifies candidate genes whose expression shifts in stylopized hosts, giving researchers a starting point for asking what the parasite might be secreting and what the host might be responding to. The most powerful version of that experiment would compare truly parasitized wasps, pseudo-parasitized wasps, sham controls, and naturally dispersing unparasitized females at the level of neural gene expression - asking not just whether the infected state differs from the control state, but whether it resembles a state the host can reach on its own. By contrasting the neural transcriptomes of pseudo-parasitized individuals directly against naturally expressed alternative phenotypes, we can ask whether the parasite is writing unique genomic code or merely turning a pre-existing physiological page.

Finally, natural variation in infection intensity offers a relatively accessible test. Passive manipulation predicts a threshold-like response: once infection crosses some level of physical insult sufficient to trigger the host program, additional parasite load should add little. Active manipulation, at least under some models, predicts a more graded dose-response relationship. Neither prediction is ironclad, but the contrast is clear enough to be informative.

## Discussion

The *Xenos*/*Polistes* system will remain a compelling example of parasite-associated behavioral change regardless of how the mechanism resolves. Parasitized wasps do behave in ways that benefit the parasite. But the fact that a behavioral sequence benefits a parasite does not tell us whether the parasite actively directs that sequence. The zombie metaphor is vivid, but it can smuggle mechanism into description.

A more cautious interpretation is also a more interesting one. The parasite may benefit not by inventing a new wasp, but by activating an old one in the wrong context. That possibility deserves to be treated not as a caveat, but as a hypothesis. Before we invoke puppeteers, we should test the strings.

The passive manipulation hypothesis is therefore not merely an alternative explanation for one wasp-parasite association. It is a broader caution about inference in behavioral parasitology: parasite-benefiting outcomes are not, by themselves, evidence of parasite-designed control mechanisms.

We never were able to determine the energetic cost of mounting an immune response, but we might have identified a vulnerability we hadn’t considered before.

## Data Availability

The original contributions presented in the study are included in the article/supplementary material. The recovered field-activity dataset supporting **Figure 1B**, alongside the archived figure files and analysis notes comprising the patterns in **Figures 1A** and **C**, have been deposited as a unified historical archive in the Supplementary Material. Further inquiries can be directed to the corresponding author.
